# Estradiol Uses Different Mechanisms in Astrocytes from the Hippocampus of Male and Female Rats to Protect against Damage Induced by Palmitic Acid

**DOI:** 10.3389/fnmol.2017.00330

**Published:** 2017-10-24

**Authors:** Laura M. Frago, Sandra Canelles, Alejandra Freire-Regatillo, Pilar Argente-Arizón, Vicente Barrios, Jesús Argente, Luis M. Garcia-Segura, Julie A. Chowen

**Affiliations:** ^1^Departamento de Pediatría, Universidad Autónoma de Madrid, Madrid, Spain; ^2^Departamento de Endocrinología, Hospital Infantil Universitario Niño Jesús, Madrid, Spain; ^3^Instituto de Investigación Sanitaria Princesa, Madrid, Spain; ^4^Centro de Investigación Biomédica en Red de Fisiopatología de la Obesidad y Nutrición, Instituto de Salud Carlos III, Madrid, Spain; ^5^IMDEA Food Institute, Universidad Autónoma de Madrid, Consejo Superior de Investigaciones Científicas (CSIC), Madrid, Spain; ^6^Instituto Cajal, Consejo Superior de Investigaciones Científicas (CSIC), Madrid, Spain; ^7^CIBER de Investigación Biomédica en Red de Fragilidad y Envejecimiento Saludable (CIBERFES), Instituto de Salud Carlos III, Madrid, Spain

**Keywords:** endoplasmic reticulum stress, palmitic acid, estradiol, hippocampus, astrocytes

## Abstract

An excess of saturated fatty acids can be toxic for tissues, including the brain, and this has been associated with the progression of neurodegenerative diseases. Since palmitic acid (PA) is a free fatty acid that is abundant in the diet and circulation and can be harmful, we have investigated the effects of this fatty acid on lipotoxicity in hippocampal astrocytes and the mechanism involved. Moreover, as males and females have different susceptibilities to some neurodegenerative diseases, we accessed the responses of astrocytes from both sexes, as well as the possible involvement of estrogens in the protection against fatty acid toxicity. PA increased endoplasmic reticulum stress leading to cell death in astrocytes from both males and females. Estradiol (E2) increased the levels of protective factors, such as Hsp70 and the anti-inflammatory cytokine interleukin-10, in astrocytes from both sexes. In male astrocytes, E2 decreased pJNK, TNFα, and caspase-3 activation. In contrast, in female astrocytes E2 did not affect the activation of JNK or TNFα levels, but decreased apoptotic cell death. Hence, although E2 exerted protective effects against the detrimental effects of PA, the mechanisms involved appear to be different between male and female astrocytes. This sexually dimorphic difference in the protective mechanisms induced by E2 could be involved in the different susceptibilities of males and females to some neurodegenerative processes.

## Introduction

Astrocytes are the most abundant cell type in the central nervous system and are essential to maintain its homeostasis. One important function of these glial cells is to protect neurons (Sofroniew, 2005) and they are not only involved in the pathogenesis of neurodegenerative diseases (Maragakis and Rothstein, 2006), but also in metabolic disorders such as obesity (García-Cáceres et al., 2012). Prolonged poor dietary habits can result in hypothalamic inflammation and astrogliosis (De Souza et al., 2005; Horvath et al., 2010), with more recent studies suggesting that other brain areas may also be affected (White et al., 2009; Shefer et al., 2013). Plasma concentrations of free fatty acids (FFA) are elevated in obese subjects (Arner and Ryden, 2015) and this can induce lipotoxicity, resulting in cell damage and the disruption of cellular homeostasis due to oxidative stress (de Morentin et al., 2010).

Excess intake of saturated fatty acids can affect energy homeostasis and body weight regulation and has also been associated with increased cognitive impairment (Stranahan et al., 2011). Diet-induced metabolic dysfunction has been shown to lead to brain inflammation and reactive gliosis in both experimental animals and humans (De Souza et al., 2005; Elias et al., 2005; Waldstein and Katzel, 2005; Horvath et al., 2010; Pistell et al., 2010). As the brain is highly sensitive to inflammatory mediators, this diet-induced increase in pro-inflammatory cytokines can have harmful effects on cognition and neuronal homeostasis (McAfoose and Baune, 2009). Moreover, high fat diets increase the uptake of fatty acids into the brain (Karmi et al., 2010), with some studies indicating that saturated fatty acids accumulate mainly in astrocytes (Morand et al., 1979; Bernoud et al., 1998). In addition, membrane phospholipids are degraded after traumatic or hypoxic injuries in the brain resulting in the release of FFAs, such as palmitic acid (PA) (White et al., 2000; Park et al., 2011). PA can inhibit the insulin signaling pathway, which induces endoplasmic reticulum stress in hypothalamic neurons (Mayer and Belsham, 2010). This fatty acid also up-regulates BACE1 with the consequent amyloidogenic processing of beta-amyloid precursor protein in primary cortical neurons by elevating oxidative stress and FFA metabolism in astrocytes (Patil et al., 2007) and PA-induced lipotoxicity induces apoptotic cell death in some cell types (Maestre et al., 2003; Martins de Lima et al., 2006; Ricchi et al., 2009). However, PA-induced lipotoxicity has not been extensively studied in astrocytes.

The lipotoxic effect of PA involves elevated oxidative stress and endoplasmic reticulum stress, and can result in apoptotic cell death (Ulloth et al., 2003; Almaguel et al., 2009). The endoplasmic reticulum responds to cellular insults, including metabolic insults associated with obesity, inducing the expression of genes that control cell survival. Indeed, it has been postulated that obesity is a chronic stimulus for endoplasmic reticulum stress in peripheral tissues (Özcan et al., 2004). Cells respond to mild endoplasmic reticulum stress by increasing the production of chaperones (Szegezdi et al., 2006), but when they are subjected to intense or prolonged endoplasmic reticulum stress, apoptosis is induced to eliminate the damaged cells to protect the organism (Ron, 2002). In this context, estrogens exert beneficial physiological effects, such as the attenuation of oxidative and endoplasmic reticulum stress (Behl et al., 1997). The protective actions of estrogens are mediated by the activation of at least four different receptors, all of which are expressed in astrocytes (Fuente-Martin et al., 2013; Acaz-Fonseca et al., 2016). However, the sequential effects of estrogens at the molecular level on these two stresses in astrocytes due to saturated FFA overload remain unknown. To address this issue, we have investigated PA-induced inflammation and endoplasmic reticulum stress in rat hippocampal astrocyte and the protective effects of estradiol (E2). Moreover, as males and females differ in their response to metabolic signals (Shi and Clegg, 2009) and their propensity to develop cognitive problems (Roof and Hall, 2000; Cahill, 2006), we have analyzed whether there is a sexual dimorphism in the response of hippocampal astrocytes to these factors.

## Materials and Methods

### Materials

Electrophoresis reagents were from BioRad Laboratories (Hercules, CA, United States) and the rest of chemicals and reagents were purchased from Sigma or Merck (Barcelona, Spain) unless otherwise indicated.

### Cell Cultures and Treatment

All procedures have been carried out in accordance with the local ethics committee of Animal Experimentation of the Instituto Cajal (CEEA-IC) and the Comunidad de Madrid, Reference PROEX 112/15 and complied with Royal Decree 53/2013 pertaining to the protection of experimental animals and with the European Community Council Directive (2010/63/EU).

Primary astrocytes were derived from male and female 2 day-old Wistar rat pups as described previously (Fuente-Martin et al., 2012). Briefly, primary cultures were generated from the hippocampus and maintained in DMEM:F12 (Gibco) with 10% fetal bovine serum (FBS). After 10 days *in vitro*, microglia and oligodendrocytes were removed from 70 to 80% confluent astrocyte cultures by orbital rotation for at least 16 h at 280 rpm at 37°C on an orbital shaker. The remaining cells were plated at a density of 1.5 × 10^4^ cells/cm^2^ and allowed to recover for 24 h. They were then grown in serum free media for 24 h before treatment. Fatty acid stock solutions of 200 mM were prepared in 100% EtOH. Working solutions of 5 mM fatty acids were made by incubating the fatty acids in media containing 10% endotoxin and fatty acid free BSA at 37°C for 30–60 min with occasional vortexing. This solution was then added to cells to obtain the final fatty acid concentrations. The fatty acid-albumin molar ratio was kept at <3 to ensure that the fatty acids were bound to albumin (Svedberg et al., 1990). The final concentration of solvent (ethanol) in the medium was 0.25% (v/v) for 0.50 mM PA and 0.1% (v/v) for 10^-10^ M E2. Equal volumes of the medium/EtOH/BSA vehicle were applied to control cells. In addition, 50 μl of 200 mM carnitine per 10 ml of media was added to all fatty acid treatments. Astrocytes were treated for 24 h with different doses of PA to establish a dose-response curve. Astrocytes were treated for 15 min or 2 h with 0.5 mM PA to study extracellular signal–regulated kinase (ERK), c-Jun N-terminal kinase (JNK), p38, and Akt activation. The effect of 17β E2 (Sigma) on PA was studied by adding 0.5 mM PA in combination with E2 prepared in ETOH at 10^-10^ M in the presence or absence of PA 0.5 M.

Another set of experiments was performed by pretreatment with E2 (10^-8^ or 10^-10^ M) for 4 h and then incubated with PA at 0.25 or 0.5 M for 20 h.

To verify the involvement of endoplasmic reticulum stress in the response to PA, the inhibitor 4-phenylbutyrate (4-PBA) was employed. It was dissolved in ETOH and added to astrocyte cultures at a concentration of 5 mM. The final concentration of solvent was 0.1% (v/v).

### Real Time Polymerase Chain Reaction (PCR)

Total RNA was extracted following the instructions of RNeasyPlus Mini kit (Qiagen, Hilden, Germany). Absorbance at 260 was measured to determine concentrations. cDNA was synthesized from 1.5 μg of total RNA by using a high capacity cDNA reverse transcription kit (Applied Biosystems, Foster City, CA, United States). Quantitative real-time PCR was performed by using assay-on-demand kits (Applied Biosystems) (Supplementary Table 1). TaqMan Universal PCR Master Mix (Applied Biosystems) was used according to the manufacturer’s protocol in an ABI PRISM 7000 Sequence Detection System (Applied Biosystems) with conventional parameters (95°C for 10 min, 40 cycles of 95°C for 15 s, and 60°C for 1 min). Each sample was run in duplicate and was normalized to the housekeeping gene GAPDH. According to the manufacturer’s guidelines, the ΔΔ*C*_T_ method was used to determine relative expression levels. Statistics were performed using ΔΔ*C*_T_ values.

To determine IL-6, TNF-α, and IL-10 mRNA levels, RT^2^ First Strand Kit and RT^2^ qPCR Primer assay (Qiagen, Madrid, Spain) were used for better accuracy of these genes and normalized to the housekeeping gene GAPDH (Supplementary Table 1).

### Protein Purification and Quantification

The supernatant collected in the RNA extraction process was diluted in acetone and frozen, samples were then centrifuged. Proteins were re-suspended in 100 μl of CHAPS buffer, containing 7 M urea, 2M thiourea, 4% CHAPS and 0.5%. Tris 1M pH 8.8. Protein concentration was measured using the BioRad Protein Assay (BioRad).

### Immunoblotting

Proteins were resolved using 8–12% SDS-PAGE and transferred onto PVDF membranes. Filters were blocked with TBS with 0.1% (v/v), Tween 20, and 5% (w/v) BSA or non-fat dried milk and incubated overnight at 4°C with the primary antibody in blocking buffer. Primary antibodies used are shown in Supplementary Table 2. Filters were washed and incubated with the corresponding secondary antibodies conjugated with peroxidase at a dilution of 1:2000 (Pierce, Rockford, IL, United States). Bound peroxidase activity was visualized by immune-Clarity Western Chemiluminiscent substrate (BioRad) and quantified by densitometry using a ImageQuant LAS 4000 mini system (GE Healthcare Little Chalfont, United Kingdom). All data were normalized to control values on each membrane.

### Cell Death Detection ELISA

This assay was carried out according to the manufacturer’s instructions (Roche Diagnostics, Mannheim, Germany). Briefly, tissue was homogenized in incubation buffer and microtiter plates were coated with anti-histone antibody. The samples were added (in duplicate) and incubated (90 min at room temperature). The wells were washed and incubated with anti-DNA-peroxidase (90 min at room temperature). After washing, substrate solution was added until the color developed adequately (approximately 15 min). The samples were measured at 405 nm on an automatic microplate analyzer (Tecan Infinite M200, Grödig, Austria). Background measurements at 490 nm were made and this value subtracted from the mean value of each sample.

### Crystal Violet Assay

The cells were grown and differentiated in 24-well culture dishes and after 24 h of treatment, the media was removed and the cells fixed with 1% glutaraldehyde for 20 min at 25°C. After washing with phosphate-buffered saline (PBS), 0.1% crystal violet was added to each well for 20 min at 25°C. The wells were then washed under running water for 20 min. After drying, 2 ml of 10% acetic acid was added to each well. The intensity of the resulting color was measured at 590 nm on an automatic microplate analyser (Infinite M200 TECAN, Grödig, Austria).

### Immunofluorescence

For immunocytochemistry assays, cells were plated over 12-mm diameter cover glasses in 24-well culture dishes and grown as described above. Following the corresponding treatments, the cells were washed twice with 0.1 M PBS (pH 7.4) and fixed with 4% paraformaldehyde for 15 min. After two washes with PBS, washing buffer (0.1% bovine serum albumin, 0.1% Triton-X100 in PBS) was added to equilibrate the cells for 15 min before incubating for 2 h at room temperature with blocking buffer (3% bovine serum albumin, 1% Triton-X100 in PBS). The cells were incubated overnight at 4°C in a humidified chamber with an immunocytochemistry specific primary antibody for glial fibrillary acidic protein (GFAP; 1:500; Sigma). After washing three times, the cells were incubated for 1 h at room temperature with an Alexa Fluor 488 goat anti-mouse IgG antibody (1:2000; Molecular Probes, Eugene, OR, United States) in blocking buffer. All cells were washed three times in washing buffer before the coverslips were placed over slides after adding Clear-Mount (Electron Microscopy Sciences, Hartfield, PA, United States) containing 5 mM DRAQ5 (Thermo Scientific) for nuclear staining. Finally, immunofluorescence was visualized with a confocal scanner Leica TCS SL installed in a Leica DM IRB microscope (Leica, Wetzlar, Germany). Control experiments omitting the primary antibody were performed in all assays and no specific labeling was found.

### Statistical Analysis

All data are shown as mean ± SEM. Three-way or two ANOVAs were performed to analyze the effects of the sex and treatments. When significant effects were found, a one-way ANOVA followed by Bonferroni’s *post hoc* test was used to determine differences between treatment groups. Statistical significance for all analyses was accepted at *p* < 0.05. Statistical analyses were performed using Statview 5.0.1 (SAS Institute Inc., Cary, NC, United States) and Prisma software 6.0 (Prisma, GraphPad, San Diego, CA, United States).

## Results

### Effects of PA on Intermediate Filaments of Astrocytes

**FIGURE 1:**
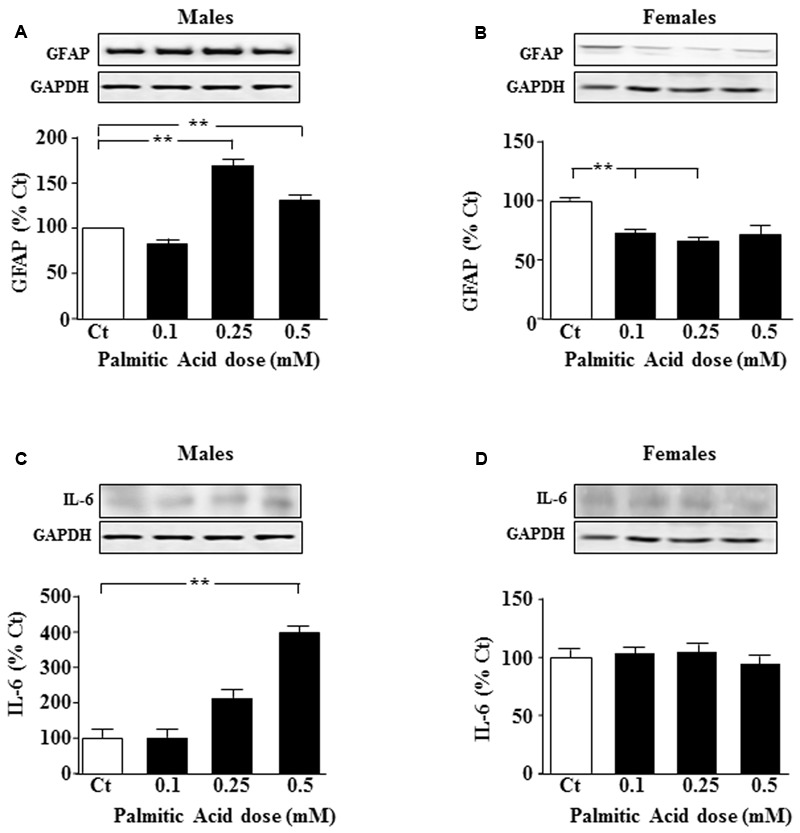
Palmitic acid (PA) dose-response curve at 24 h. Astrocyte cultures were treated with doses of 0.1, 0.25, and 0.5 mM of PA. Immunoblots were probed with antibodies toward glial fibrillary acidic protein (GFAP) in male **(A)**, and female **(B)** astrocytes and interleukin 6 (IL-6) in male **(C)** and female **(D)** astrocytes. The average of three independent assays performed in duplicate is shown. Statistical significance: ^∗∗^*p* < 0.01.

Primary astrocyte cultures were treated for 24 h with different doses of PA and the effect on specific intermediate filaments was studied. In males, GFAP levels were increased in response to 0.25 and 0.5 mM PA (*P* < 0.05; **Figure 1A**); however, in females at the two lower doses tested, a decrease in GFAP protein levels was observed (*P* < 0.01; **Figure 1B**).

### Effects of PA on Inflammatory Factors

To test whether inflammatory signals were affected, levels of interleukin (IL)-6 and p-IkappaB were measured after addition of different doses of PA. IL-6 levels increased in males after addition of 0.5 mM PA (*P* < 0.01; **Figure 1C**). PA had no effect on IL-6 protein levels in female astrocytes (**Figure 1D**).

Levels of the pro-inflammatory intracellular signal, p-IkappaB did not change in male or female astrocytes after PA treatment (data not shown).

### Effects of PA on MAPK and Akt Pathways

**FIGURE 2:**
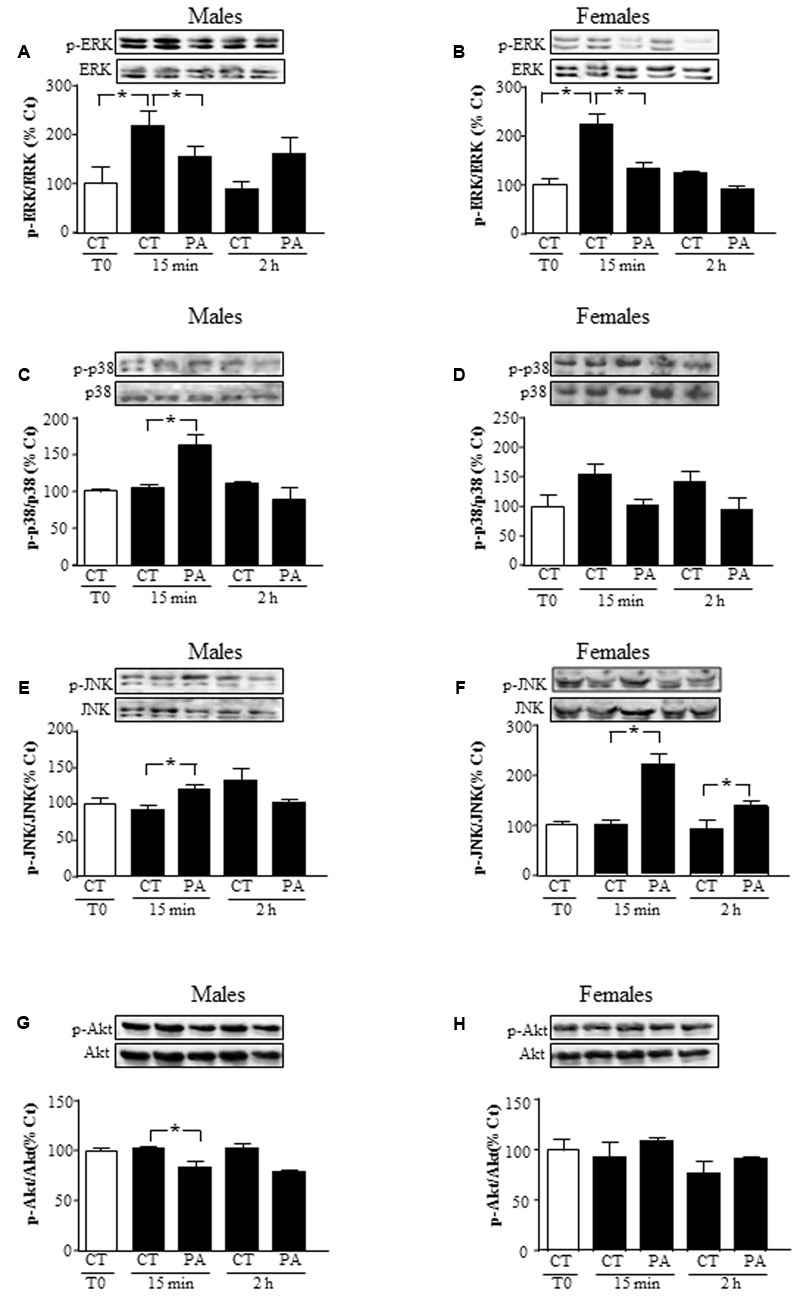
Effects of PA on the activation of mitogen activated protein kinases (MAPKs) and protein kinase B (Akt). Astrocyte cultures were treated with PA (0.5 mM), E2 (10^-10^ M) or the combination of both. Immunoblots were probed with antibodies toward phosphorylated-extracellular signal-regulated kinases (pERKs) and extracellular signal-regulated kinases (ERKs) in male **(A)**, and female **(B)** astrocytes; phosphorylated-p38 (p-p38) and p38 in male **(C)** and female **(D)** astrocytes; phosphorylated (p)-c-Jun N-terminal kinase (p-JNK) and JNK in astrocyte cultures of males **(E)** and females **(F)** and phosphorylated-protein kinase B (p-Akt) and protein kinase B (Akt) in male **(G)**, and female **(H)** astrocytes. The average of three independent assays performed in duplicate is shown. Statistical significance: ^∗^*p* < 0.05.

We characterized whether astrocytes activated the kinases ERK, p38 MAPK, JNK, and Akt in response to PA. PA (0.5 mM) decreased mitogenic ERK activation in astrocytes of both sexes at 15 min (*P* < 0.05; **Figures 2A,B**, respectively). In male astrocytes, increased activation of inflammatory-related kinases p38 and JNK was found at 15 min, but not at 2 h (*P* < 0.05; **Figures 2C,E**). In females, activation of JNK was found at 15 min and 2 h, with no significant change in p38 activation at 15 min or 2 h (*P* < 0.05; **Figures 2D,F**).

Levels of phosphorylated Akt, a survival related kinase, decreased in astrocytes from males at 15 min (*P* < 0.05), but did not change in those from females at 15 min or 2 h (**Figures 2G,H**).

### Actions of E2 on the Effects of PA

**FIGURE 3:**
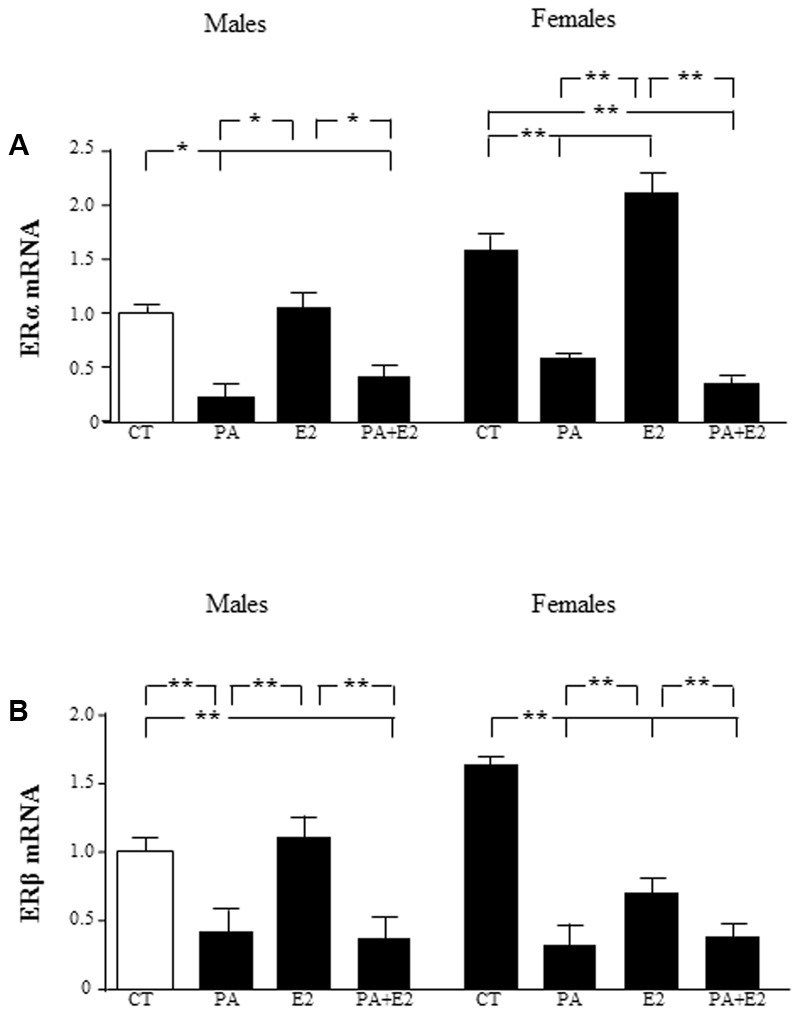
Effects of PA and 17β estradiol (E2) on estrogen receptor α (ERα) and estrogen receptor β (ERβ) mRNA levels. Astrocyte cultures were treated with PA (0.5 mM), E2 (10^-10^ M) or the combination of both. Relative mRNA levels of ERα in cultures from males and females **(A)**; and ERβ in cultures from males and females **(B)** were measured. The average of three independent assays performed in duplicate is shown. Statistical significance: ^∗^*p* < 0.05 and ^∗∗^*p* < 0.01.

Estrogenic hormones play a protective role in inflammatory situations (Azcoitia et al., 2011). This prompted us to investigate whether E2 could prevent PA induced inflammation in astrocytes. We first assessed the effect of the co-treatment with PA and E2 on the levels of estrogen receptors α (ERα) and β (ERβ) mRNA. There was an effect of sex [*F*_(1,40)_: 39.9; *P* < 0.001] and PA [*F*_(1,40)_: 224.8; *P* < 0.001] on ERα mRNA levels with interactions between sex and PA [*F*_(1,40)_: 34.7; *P* < 0.001], sex and E2 [*F*_(1,40)_: 11.5; *P* < 0.01] and sex, PA and E2 [*F*_(1,40)_: 8.4; *P* < 0.01]. PA decreased the mRNA levels of ERα in male and female astrocytes. E2 increased the mRNA levels of ERα only in astrocytes from females, but the addition of E2 was unable to block the PA induced decrease in the mRNA levels of ERα in neither males nor females (**Figure 3A**).

There was an effect of PA [*F*_(1,40)_: 138.5; *P* < 0.001] on ERβ mRNA levels with interactions between PA and E2 [*F*_(1,40)_: 4.9; *P* < 0.05] and sex, PA and E2 [*F*_(1,40)_: 7.6; *P* < 0.01]. The mRNA levels of ERβ were also decreased by PA in both male and female astrocytes. However, E2 decreased the mRNA levels of ERβ only in female astrocytes. The co-treatment of PA and E2 decreased the mRNA levels of ERβ in males and females (**Figure 3B**).

**FIGURE 4:**
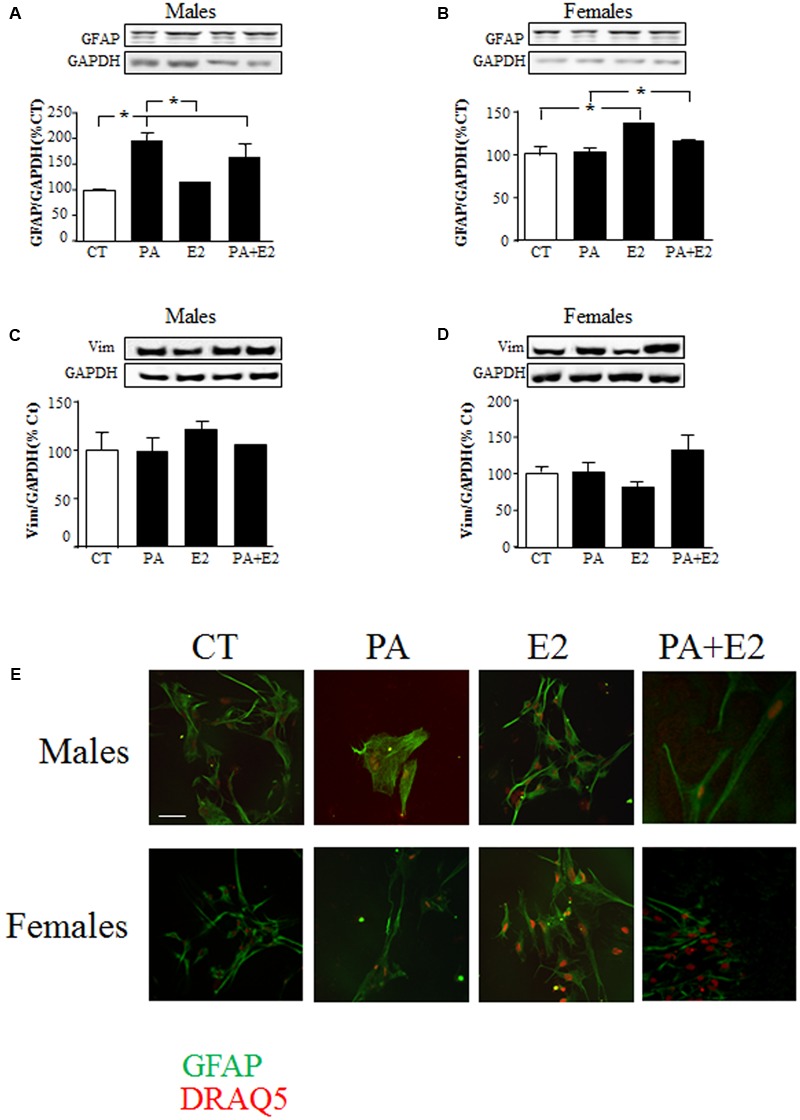
Effects of PA and 17β E2 on astrocytes markers. Astrocyte cultures were treated with PA (0.5 mM), E2 (10^-10^ M) or the combination of both. Immunoblots were probed with antibodies toward GFAP in males **(A)**, and females **(B)**; Vimentin in males **(C)**; and females **(D)**. The average of three independent assays performed in duplicate is shown. Statistical significance: ^∗^*p* < 0.05. Immunocytochemistry for GFAP and nuclear DRAQ5 staining **(E)**. Scale bar, 50 μm.

There was an effect of PA on GFAP protein levels in males [*F*_(1,20)_: 51.7; *P* < 0.001] with an interaction between PA and E2 [*F*_(1,20)_: 6.3; *P* < 0.05]. As in the previous study PA increased GFAP levels in male astrocytes, while E2 had no effect either alone or in combination with PA (**Figure 4A**). In females, there was an effect of PA [*F*_(1,20)_: 12.8; *P* < 0.005] and of E2 [*F*_(1,20)_: 39.3; *P* < 0.001] on GFAP levels. E2 increased GFAP levels (*P* < 0.05; **Figure 4B**) and although PA alone had no effect, it reduced the stimulatory effect of E2 (*P* < 0.05 vs. PA; **Figure 4B**). E2 had no effect on vimentin levels in males or females (**Figures 4C,D**).

Immunohistochemistry for GFAP was performed to observe the morphology of cells in response to the treatments. Male astrocytes treated with PA alone had a hypertrophic cell body and processes (**Figure 4E**). PA did not affect the morphology of female astrocytes. No effect of E2 was observed in either sex.

### Effects of PA and E2 on Proliferation and Cell Death

**FIGURE 5:**
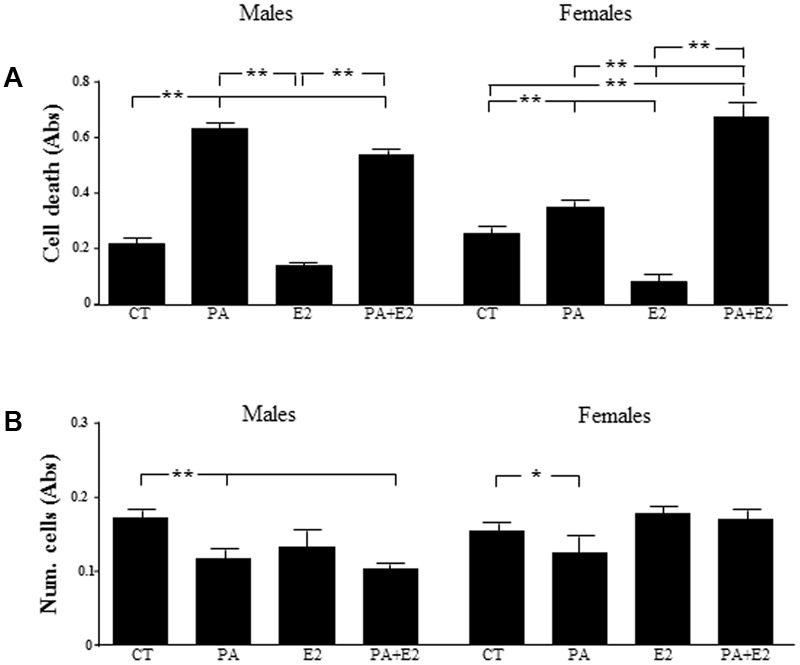
Effects of PA and 17β E2 on cell death and cell viability. Astrocyte cultures were treated with PA (0.5 mM), E2 (10^-10^ M) or the combination of both. Cell death was assayed by ELISA in males **(A)** and females **(B)**. Viability was assayed by crystal violet. The average of three independent assays performed in triplicate is shown. Statistical significance: ^∗^*p* < 0.05 and ^∗∗^*p* < 0.01.

The role of PA and E2 on the balance between proliferation and cell death was analyzed. There was an effect of PA [*F*_(1,24)_: 219.0; *P* < 0.001] on cell death and on cell number [*F*_(1,24)_: 37.6; *P* < 0.001]: For cell death there was interactions between sex and E2 [*F*_(1,24)_: 4.6; *P* < 0.05], PA and E2 [*F*_(1,24)_: 33.5; *P* < 0.001] and sex, PA and E2 [*F*_(1,24)_: 7.8; *P* < 0.05]. There was an interaction between sex and E2 [*F*_(1,24)_: 6.6; *P* < 0.05]; and between PA and E2 [*F*_(1,24)_: 7.7; *P* < 0.05] on cell number. PA increased cell death in male and female astrocyte cultures (**Figure 5A**) and reduced the overall number of cells (**Figure 5B**). In males, E2 had no effect on cell death or cell number, either alone or in combination with PA. In females, E2 decreased basal astrocytic cell death but was unable to protect against the PA induced cell death; moreover, the combination of PA and E2 increased cell death (**Figure 5A**). Although E2 alone had no effect on cell number in females, in combination with PA it was able to maintain the number of cells such that they were not different from control levels (**Figure 5B**).

**FIGURE 6:**
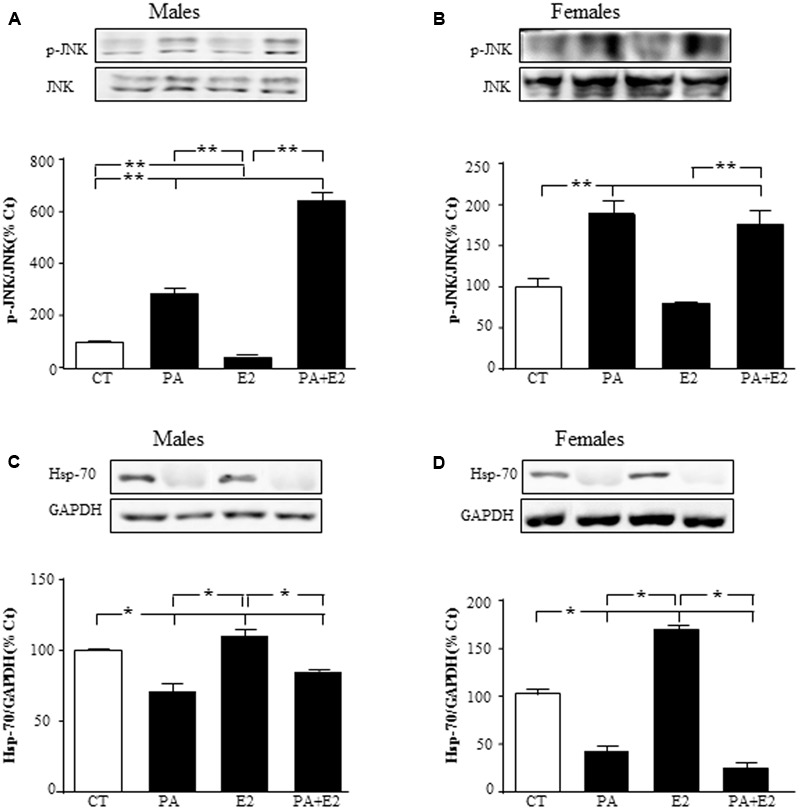
Effects of PA and 17β E2 on c-Jun N-terminal kinase (JNK) and heat shock protein 70 (Hsp70). Astrocyte cultures were treated with PA (0.5 mM), E2 (10^-10^ M) or the combination of both. Immunoblots were probed with antibodies toward phosphorylated-(p-JNK) and JNK in males **(A)**, and females **(B)**; and Hsp70 in males **(C)**; and females **(D)**. The average of three independent assays performed in duplicate is shown. Statistical significance: ^∗^*p* < 0.05 and ^∗∗^*p* < 0.01.

We then studied the effect of E2 on the inflammatory related kinase, JNK, at 24 h and observed an effect of PA on JNK in males [*F*_(1,20)_: 76.6; *P* < 0.001] and in females [*F*_(1,20)_: 73.1; *P* < 0.001] with PA increasing the activation of JNK in both sexes. There was also an effect of E2 [*F*_(1,20)_: 7.5; *P* < 0.05] and an interaction between PA and E2 on JNK in males [*F*_(1,20)_: 15.5; *P* < 0.05]. In males, E2 alone reduced the activation of JNK; however, not only was this sex steroid unable to block the PA induced increase in pJNK levels, but the combination further increased its activation (**Figure 6A**). In females, PA increased pJNK levels at 24 h of treatment; however, E2 had no effect either alone or in combination with PA (**Figure 6B**).

Heat shock protein p70 (Hsp70) can prevent or arrest inflammatory damage. There was an effect of PA in males [*F*_(1,20)_: 111.1; *P* < 0.001] and in females [*F*_(1,20)_: 88.8; *P* < 0.001], with this fatty acid reducing Hsp70 in astrocyte cultures of both sexes (**Figures 6C,D**). Hsp70 was also affected by E2 in both males [*F*_(1,20)_: 22.6; *P* < 0.001] and females [*F*_(1,20)_: 16.8; *P* < 0.01], with an interaction between PA and E2 [*F*_(1,20)_: 32.6; *P* < 0.001] in females. Some protective effects of E2 in brain are mediated by inducing the expression of Hsp70 (Zhang et al., 2014); in agreement with this, E2 increased the levels of Hsp70 in both male and female astrocytes (**Figures 6C,D**). However, E2 was unable to impede the decrease in Hsp70 levels induced by PA in both sexes.

### Effects of PA and E2 on Pro- and Anti-inflammatory Cytokines

**FIGURE 7:**
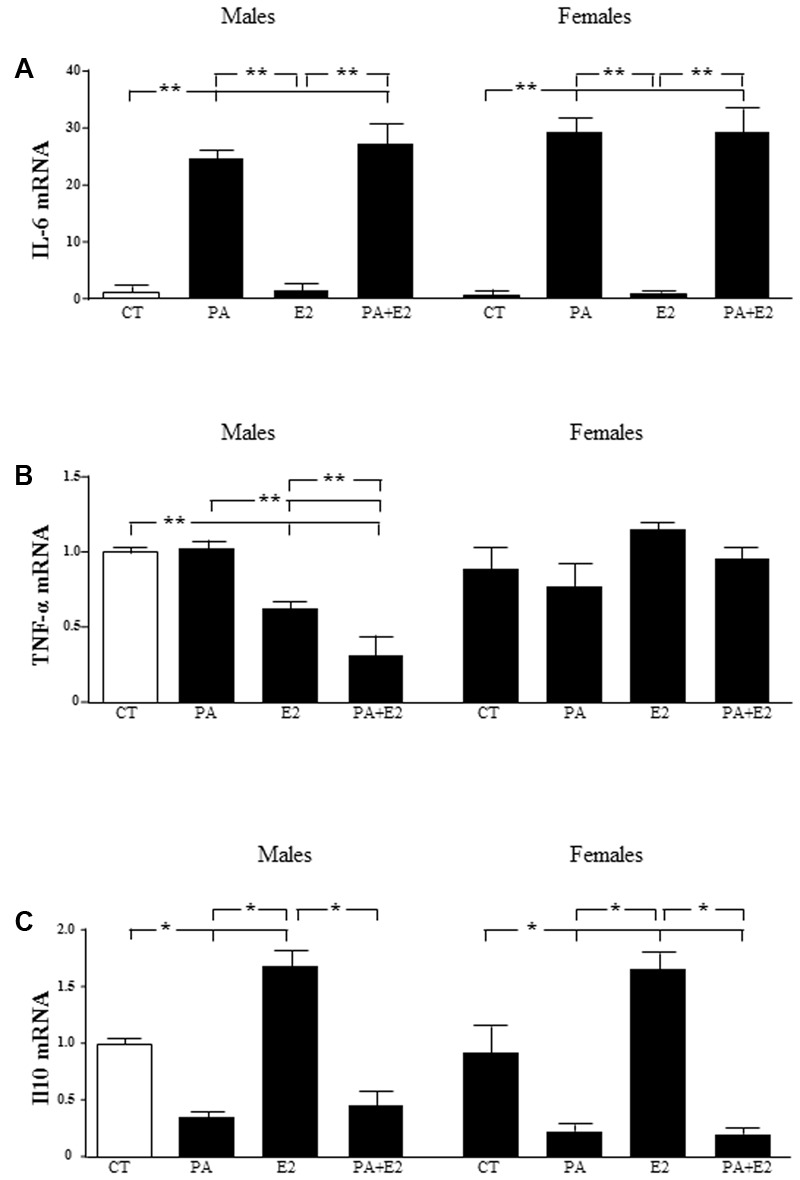
Effects of PA and 17β E2 on cytokine mRNA levels. Astrocyte cultures were treated with PA (0.5 mM), E2 (10^-10^ M), or the combination of both. Relative mRNA levels of IL-6 in males and females **(A)**; tumor necrosis factor α (TNFα) in males and females **(B)** and IL-10 in males and females **(C)** were measured. The average of three independent assays performed in duplicate is shown. Statistical significance: ^∗^*p* < 0.05 and ^∗∗^*p* < 0.01.

There was an effect of PA [*F*_(1,40)_: 119.8; *P* < 0.001] on IL6 mRNA levels, with PA inducing an increase in both male and female astrocytes. E2 had no effect on this parameter in either sex and was unable to reverse the increase induced by PA (**Figure 7A**).

There was an effect of sex [*F*_(1,40)_: 8.3; *P* < 0.01] and E2 [*F*_(1,40)_: 8.3; *P* < 0.05] on TNF-α mRNA levels, with an interaction between sex and E2 [*F*_(1,40)_: 50.4; *P* < 0.001]. The mRNA levels of TNF-α were not affected by PA in either males or females. In contrast, E2 alone reduced TNF-α mRNA levels in males and this decrease was greater in combination with PA. There was no effect in females (**Figure 7B**).

There was an effect of PA [*F*_(1,40)_: 52.3; *P* < 0.001] and of E2 [*F*_(1,40)_: 7.2; *P* < 0.05] on IL10 mRNA levels with an interaction between PA and E2 PA [*F*_(1,40)_: 5.7; *P* < 0.05]. PA decreased the mRNA levels of IL10 in both male and female astrocytes. Although, E2 alone increased IL10 mRNA levels in both sexes; it did not reverse the effect of PA in either sex (**Figure 7C**).

### Effects of PA and E2 on Endoplasmic Reticulum Stress

**FIGURE 8:**
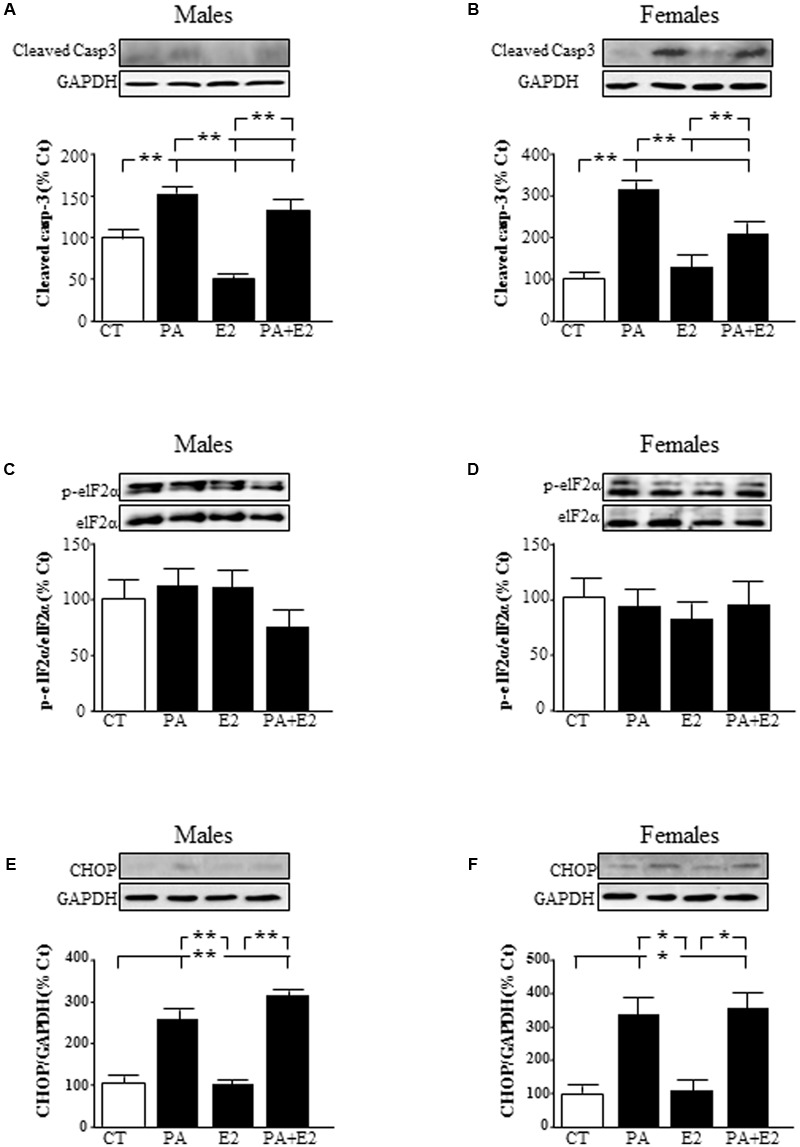
Effects of PA and 17β E2 on endoplasmic reticulum stress protein levels. Astrocyte cultures were treated with PA (0.5 mM), E2 (10^-10^ M), or the combination of both. Immunoblots were probed with antibodies toward cleaved caspase-3 levels (cleaved casp-3) in male **(A)** and female cultures **(B)**; phospho-eukaryotic initiation factor (eIF)2α and eIF2α in males **(C)** and females **(D)** and C/EBP homologous protein (CHOP) in males **(E)** and females **(F)**. The average of three independent assays performed in duplicate is shown. Statistical significance: ^∗^*p* < 0.05 and ^∗∗^*p* < 0.01.

To elucidate the molecular mechanisms involved in the toxic effects of PA, we analyzed the involvement of caspase 3 and the induction of endoplasmic reticulum stress markers. There was an effect of PA in males [*F*_(1,20)_: 223.3; *P* < 0.001] and in females [*F*_(1,20)_: 134.0; *P* < 0.001] and of E2 in males [*F*_(1,20)_: 47.9; *P* < 0.001] and in females [*F*_(1,20)_: 9.0; *P* < 0.01] on cleaved caspase-3 levels. Also there was an interaction between PA and E2 in both sexes [males: *F*_(1,20)_: 16.2; *P* < 0.01 and females: *F*_(1,20)_: 31.9; *P* < 0.001]. PA increased the activation of caspase-3, as detected by the presence of the active fragment (**Figures 8A,B**), in astrocytes from both sexes. In males, E2 alone reduced the activation of caspase-3 (**Figure 8A**), with no effect in females. However, E2 reduced the PA induced activation of caspase-3 in both sexes (**Figures 8A,B**).

The role of endoplasmic reticulum stress was accessed by analyzing the levels of phosphorylation of eukaryotic initiation factor (eIF)2α and induction of C/EBP homologous protein (CHOP) protein levels. Phosphorylation of eIF2α did not change in males or females (**Figures 8C,D**). There was an effect of PA [*F*_(1,20)_: 595.3; *P* < 0.001] and of E2 [*F*_(1,20)_: 13.9; *P* < 0.01] in males on CHOP with an interaction between PA and E2 [*F*_(1,20)_: 14.1; *P* < 0.01]. In females, there was an effect of PA [*F*_(1,20)_: 269.7; *P* < 0.001] on CHOP levels. The levels of CHOP were increased by PA in astrocytes from males (**Figure 8E**) and females (**Figure 8F**). E2 had no effect on CHOP alone or in combination with PA in astrocytes from either sex.

**FIGURE 9:**
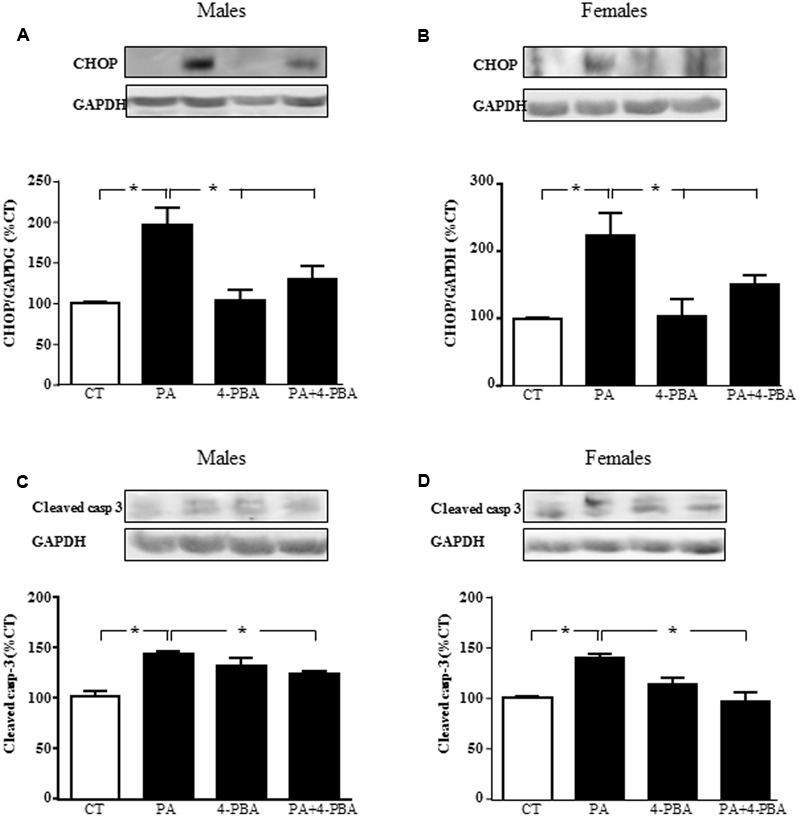
Effects of PA and 4-phenylbutyrate (4-PBA) on C/EBP homologous protein (CHOP) and cleaved caspase-3 protein levels. Astrocyte cultures were treated with PA (0.5 mM), 4-PBA (5 mM) or the combination of both. Immunoblots were probed with antibodies toward cleaved caspase-3 levels (cleaved casp-3) in male **(A)** and female **(B)** astrocyte cultures; and CHOP in males **(C)** and females **(D)**. The average of three independent assays performed in duplicate is shown. Statistical significance: ^∗^*p* < 0.05.

To verify that endoplasmic reticulum stress is involved in PA-induced cell death, astrocytes were treated with PA in combination with the endoplasmic reticulum stress inhibitor 4-PBA and levels of CHOP and cleaved caspase-3were assayed. There was an effect of PA in males [*F*_(1,12)_: 68.2; *P* < 0.001] and in females [*F*_(1,12)_: 39.2; *P* < 0.001] on CHOP, with an interaction between PA and the inhibitor in both sexes [*F*_(1,12)_: 22.6; *P* < 0.001 in males and *F*_(1,12)_: 5.2; *P* < 0.05 in females]. PA increased the levels of CHOP in both sexes. Although 4-PBA alone had no effect on CHOP levels, it blocked the increase induced by PA in males (**Figure 9A**) and females (**Figure 9B**).

There was an effect of PA in males [*F*_(1,12)_: 52.4; *P* < 0.001] and in females [*F*_(1,12)_: 11.9; *P* < 0.005] on cleaved caspase-3, with an interaction between PA and the inhibitor in both sexes [*F*_(1,12)_: 52.4; *P* < 0.0001 in males and *F*_(1,12)_: 75.8; *P* < 0.01 in females]. PA increased the levels of cleaved caspase-3, with no effect of 4-PBA. However, this inhibitor reduced the rise in cleaved caspase-3 induced by PA in males (**Figure 9C**) and females (**Figure 9D**).

### Effects of PA and E2 on Steroidogenic Proteins

**FIGURE 10:**
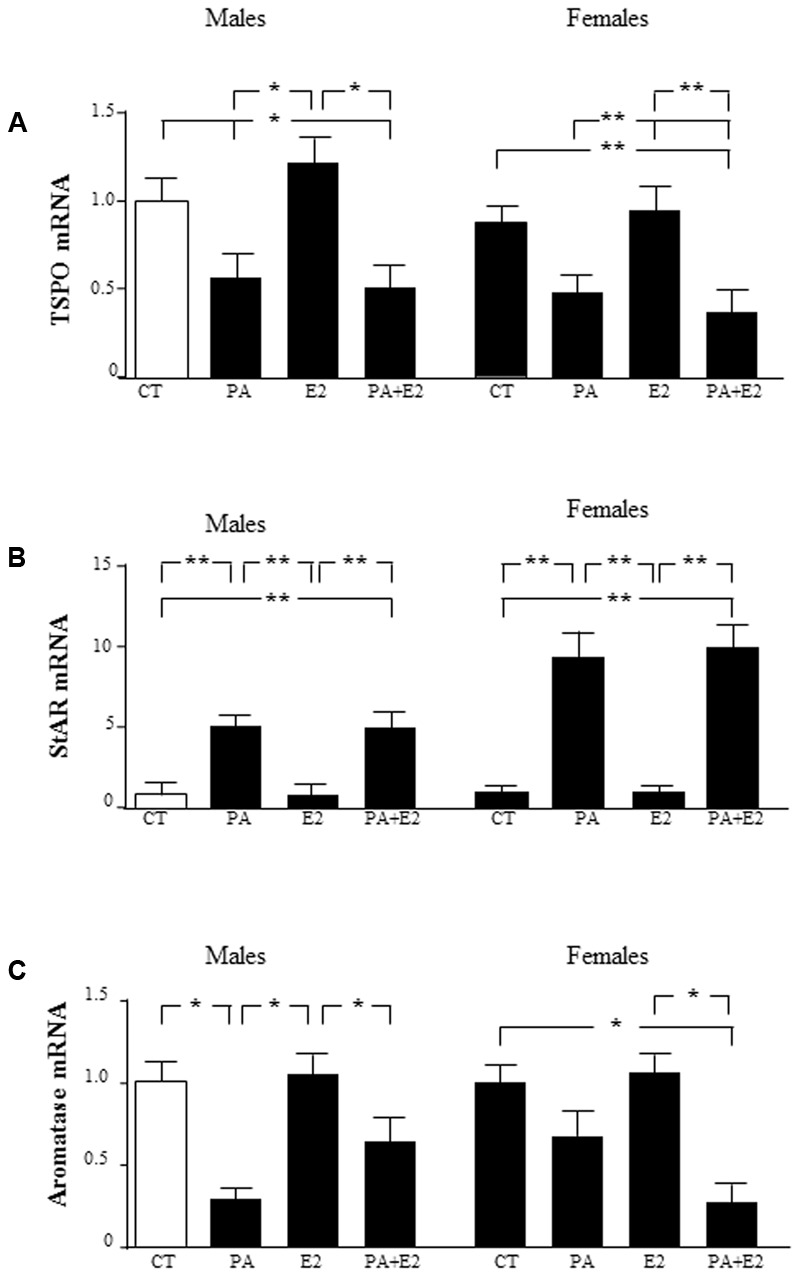
Effects of PA and 17β E2 on translocator protein (TSPO), steroidogenic acute regulatory protein (StAR), and aromatase mRNA levels. Astrocyte cultures were treated with PA (0.5 mM), E2 (10^-10^ M) or the combination of both. Relative mRNA levels of TSPO in males and females **(A)**; StAR in males and females **(B)**; and aromatase in males and females **(C)**. The average of three independent assays performed in duplicate is shown. Statistical significance: ^∗^*p* < 0.05 and ^∗∗^*p* < 0.01.

To determine whether the sex differences observed in the response of astrocytes to PA treatment were possibly associated with sex differences in the local production of sex steroids, the expression of proteins involved in steroidogenesis was assessed. The mRNA levels of two proteins involved in cholesterol transport into the mitochondria, translocator protein (TSPO), and steroidogenic acute regulatory protein (StAR), and of aromatase, the enzyme that converts testosterone to E2, were analyzed. There was an effect of PA [*F*_(1,40)_: 137.5; *P* < 0.001] on TSPO with an interaction between PA and E2 [*F*_(1,40)_: 4.68; *P* < 0.05], with PA decreasing the mRNA levels of TSPO either in the presence or absence of E2 (**Figure 10A**). E2 had no effect on TSPO mRNA expression in either sex. There was an effect of sex [*F*_(1,40)_: 84.3; *P* < 0.001] and of PA [*F*_(1,40)_: 421.8; *P* < 0.001] on StAR mRNA levels, with an interaction between sex and PA [*F*_(1,40)_: 84.3; *P* < 0.001]. The mRNA levels of StAR were increased by PA in both male and female astrocytes, with no effect of E2 in either sex. The co-treatment of PA and E2 increased the mRNA levels of StAR in males and females (**Figure 10B**).

There was an effect of PA [*F*_(1,40)_: 91.7; *P* < 0.001] on aromatase mRNA levels, with an interaction between sex and E2 [*F*_(1,40)_: 11.7; *P* < 0.05] and for sex, PA and E2 [*F*_(1,40)_: 9.4; *P* < 0.01]. PA decreased the mRNA levels of aromatase only in males. E2 alone did not affect the mRNA of aromatase in males or females. However, the co-treatment of PA with E2 reduced aromatase mRNA levels in females (**Figure 10C**).

### Effects of PA and E2 on Lipid Metabolism

**FIGURE 11:**
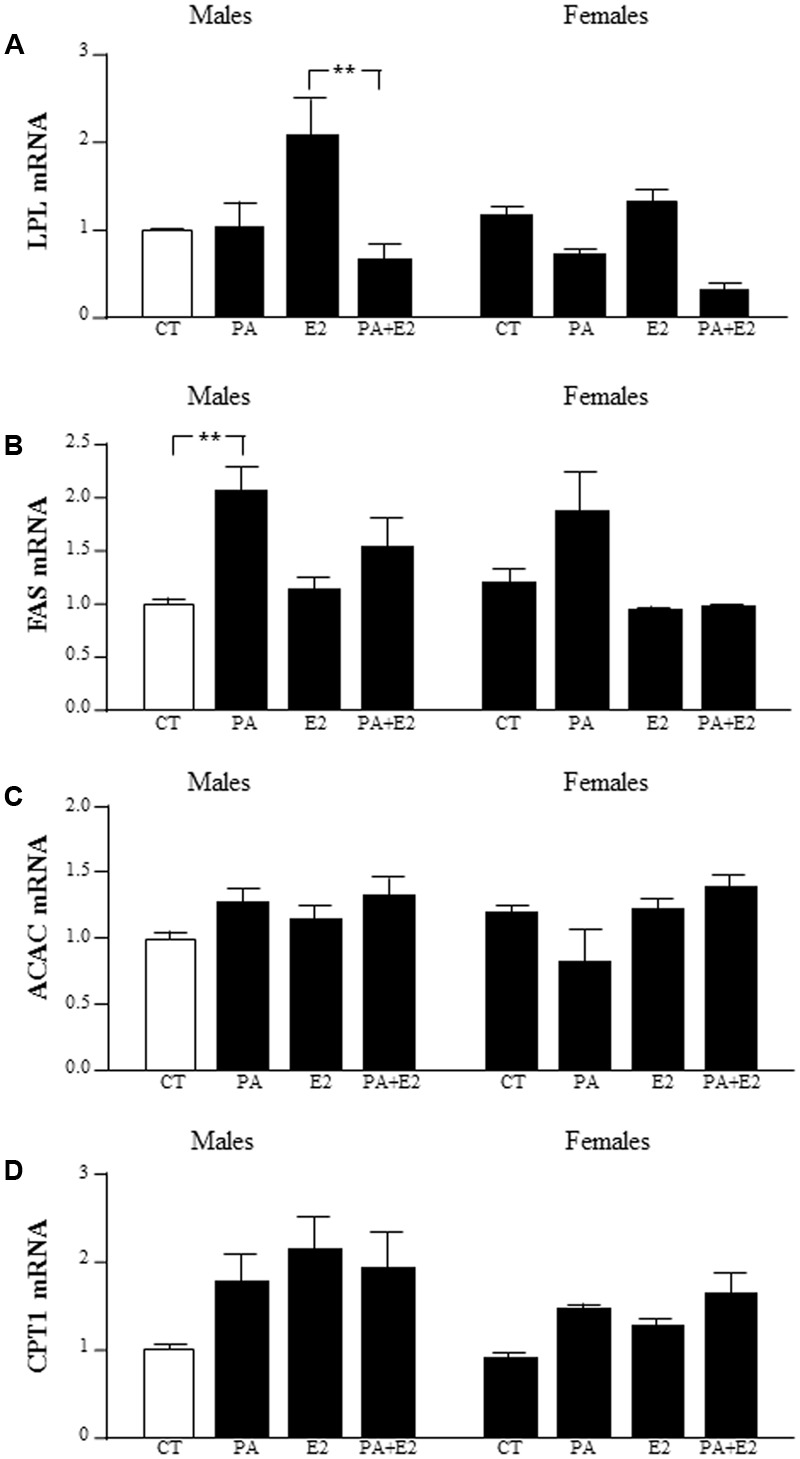
Effects of PA and 17β E2 on lipoprotein lipase (LPL), fatty acid synthase (FAS), acetyl coA carboxylase (ACAC), and carnitine palmitoyltransferase (CPT1) mRNA levels. Astrocyte cultures were treated with PA (0.5 mM), E2 (10^-10^ M) or the combination of both. Relative mRNA levels of LPL **(A)**, FAS **(B)**, ACAC **(C)**, and CPT1 **(D)** in male and female astrocyte cultures. The average of three independent assays performed in duplicate is shown. Statistical significance: ^∗∗^*p* < 0.01.

There was an effect of PA [*F*_(1,40)_: 15.6; *P* < 0.005] with an interaction between PA and E2 [*F*_(1,40)_: 7.5; *P* < 0.005] on lipoprotein lipase (LPL) mRNA levels. PA tended to decrease LPL level, with this only reaching significance in male astrocytes treated with PA+E2 compared to E2 alone (**Figure 11A**).

There was an effect of PA [*F*_(1,37)_: 12.0; *P* < 0.005] and E2 [*F*_(1,37)_: 6.1; *P* < 0.05] on fatty acid synthase (FAS) mRNA levels, with an interaction between PA and E2 [*F*_(1,37)_: 4.2; *P* < 0.05]. The mRNA levels of FAS were induced by PA in males. There was no effect in females (**Figure 11B**).

There was no effect of PA or E2 on acetyl CoA carboxylase (ACAC) mRNA levels in either sex (**Figure 11C**).

There was an effect of sex [*F*_(1,39)_: 4.1; *P* < 0.05] and E2 [*F*_(1,39)_: 5.7; *P* < 0.05] on carnitine palmitoyltransferase (CPT1A) mRNA levels, with males tending to have higher levels than females, although this did not reach statistical significance in the *post hoc* analysis (**Figure 11D**).

## Discussion

In this study, we evaluated the lipotoxic effect of PA on astrocyte cultures isolated from the hippocampus of male and female rats and our results indicate that PA reduces the viability of astrocytes in both sexes and that this lipotoxicity involves an increase in pro-inflammatory markers and an increase in endoplasmic reticulum stress that is accompanied by the activation of caspase-3. We also studied the possible protective role of E2. E2 itself had an effect on astrocyte survival in females, although it did not reverse the lipotoxic effect of PA in astrocytes from either sex. Additionally, although only the results of the experiments with co-administration of E2 and PA are presented here, experiments in which cultures were pre-incubated with E2 prior to the addition of PA were also performed and similar results were obtained. Likewise, studies using 0.25 mM PA and 10^-8^ M E2 also resulted in similar results.

An increase in saturated fatty acid intake can affect energy homeostasis and cell/tissue function (Hariri et al., 2010; Yu et al., 2010). An imbalance between energy intake and energy expenditure leads to an excess of circulating lipids and their accumulation in brain tissues (Borg et al., 2012). Excessive accumulation of FFAs in the hypothalamus is suggested to result in their redirection to non-oxidative pathways. This results in the production of toxic reactive lipid species, which can induce endoplasmic reticulum stress and lipotoxic effects that can affect the function of this brain area (Martinez de Morentin et al., 2010). The lipotoxic effect of PA is associated with elevated inflammatory mediators, endoplasmic reticulum stress and apoptotic cell death (Ulloth et al., 2003; Almaguel et al., 2009; Mayer and Belsham, 2010). However, the adverse effects of poor dietary habits and excess adipose tissue mass on brain physiology have not only been described in the hypothalamus (Maric et al., 2014), but also in the hippocampus (Bruce-Keller et al., 2009, 2010; Stranahan et al., 2011). Increased gliosis is a common finding in association with diet-induced metabolic dysfunction and neuroinflammation (White et al., 2009). Cytokines, particularly TNF-α, IL1β, and IL6, are effectors of the neuroinflammatory cascade (Allan and Rothwell, 2003) and increased levels of these cytokines can disrupt the mechanisms associated with learning and memory, which involve the hippocampus (Parnet et al., 2002). Here we found that PA reduced the viability and increased apoptosis in hippocampal astrocytes of both sexes. This was accompanied by increased pro-inflammatory IL6 expression and a reduction in the expression of anti-inflammatory IL10. Long-chain fatty acids are natural uncouplers of oxidative phosphorylation in mitochondria at low concentrations, reducing ROS by 50% (Schonfeld and Wojtczak, 2007); however, this occurs only under conditions of reverse electron transport. At higher concentrations, fatty acids can interfere with electron transport, facilitating ROS production, possibly due to the depletion of cytochrome c from mitochondria, thereby interrupting the electron flow from Complex III to IV (Di Paola and Lorusso, 2006). Such depletion has been shown to dramatically increase ROS production in heart and brain (Kushnareva et al., 2002).

In addition, lipotoxicity is reported to involve activation of the stress mediated kinases JNK and p38 in different organs and cell types (Ibrahim and Gores, 2012; Keane et al., 2015) including neurons (Mayer and Belsham, 2010) and astrocytes (Wong et al., 2014). Our results are in agreement with these previous studies as we observed activation of JNK in astrocytes from both males and females after exposure to PA. Among the molecular mechanisms involved in PA-associated apoptosis, malfunction of endoplasmic reticulum homeostasis has been postulated as a major initiator of lipid-induced toxicity (Borradaile et al., 2006). In this regard, the transcription factor CHOP plays an important role in endoplasmic reticulum stress-induced apoptosis (Li et al., 2014). The permanent up-regulation of CHOP is suggested to be decisive for the induction of astrocyte death in models of oxygen and glucose deprivation (Benavides et al., 2005). In glioblastoma cells CHOP-mediated apoptosis is reported to occur through an increase in caspase-3 in response to endoplasmic reticulum stress (Quick and Faison, 2012). CHOP induction and caspase-3 cleavage leading to apoptosis has also been described in insulinoma cells treated with PA (Simon-Szabo et al., 2014). In agreement, we found that PA increased the expression of CHOP and activation of caspase-3, as well as cell death, in both sexes. The chemical chaperone 4-PBA has been reported to reduce endoplasmic reticulum stress and consequent apoptotic signaling (Burrows et al., 2000). Here 4-PBA suppressed the PA-induced increase in CHOP and protected the astrocytes from caspase-3 activation, supporting the idea that PA-induced cell death involves endoplasmic reticulum stress. Reactive gliosis is strongly associated with brain inflammation that could be involved in the neurologic consequences of obesity (Gupta et al., 2012). However, the levels of GFAP were increased in cultures of astrocytes from males and did not change in female. This sex difference in the response of astrocytes to PA can be the result of differential regulation of the processes of proliferation, differentiation and cell death (Krebs-Kraft et al., 2010; Chen et al., 2014). Moreover, inhibition of proteasomal function that follows JNK activation contributes to GFAP accumulation (Tang et al., 2006). Differences in the development of astrocytes have been reported (McCarthy et al., 2002), including the number and morphology of astrocytes in different areas of the CNS, including the hippocampus (Garcia-Segura et al., 1988; Arias et al., 2009). Here we observed changes in the morphology of astrocytes treated with PA, with hypertrophy of the cell body and processes being observed. This morphology has been suggested to be associated with reactive astrocytes that do not proliferate (Barreto et al., 2011). Moreover, vimentin expression in our cultures did not change in response to high doses of PA; this has been reported for mild astrogliosis (Barreto et al., 2012; Acaz-Fonseca et al., 2016).

Estrogens have been described to exert physiologic functions on astrocytes that depend on the molecular environment and health status of the brain (Wise et al., 2009). E2 acts directly on astrocytes through different receptors including the classical ERα and ERβ (Azcoitia et al., 1999; Garcia-Ovejero et al., 2005; Quesada et al., 2007), as well as GPER and Gq-mER (Garcia-Ovejero et al., 2005; Almey et al., 2012). E2 promotes the growth of astrocyte processes, accompanied by an increase in GFAP expression in different brain regions including the hippocampus (Tranque et al., 1987; Stone et al., 1998).

The differential effects of E2 in the hippocampus of males and females are probably due to the different expression pattern of estrogen receptors and regulators (Tetel and Pfaff, 2010), leading to distinct cellular signaling or potency. In our study, male and female astrocytes expressed ERα and ERβ, which are known to exert a protective role against damage (Pietranera et al., 2016). In male astrocytes E2 treatment had no effect on the mRNA levels of ERα or ERβ, while in females E2 increased the mRNA levels of ERα but reduced the mRNA levels of ERβ. Other sex differences in the effect of E2 on astrocytes were also observed, such as the observation that E2 decreased the expression of TNF-α, caspase-3, and JNK in male but not in female astrocytes. In addition, E2 decreased cell death only in female astrocytes. This may be related with the increased expression of ERα in female astrocytes after E2 treatment. Although E2 increased the levels of Hsp70 in both male and female astrocytes, the effect was more evident in female astrocytes. Hsp70 has a marked cytoprotective effect and inhibits apoptotic signaling (Garrido et al., 2006) and when overexpressed it protects astrocytes against ischemic injury *in vitro* (Voloboueva et al., 2008). In muscle fibers, E2 augments Hsp70 content via ERα (Bombardier et al., 2013). Sex differences in astrocytes can result from different exposure to sex steroids during development (Arnold and Beyer, 2009) or be associated with sex chromosome differences. Previous studies have shown that perinatal actions of androgens are involved in the generation of sex differences in the expression of GFAP and in the differentiation and number of astrocytes in different brain regions (Garcia-Segura et al., 1988).

The proteins TSPO and StAR, present in astrocytes as well as other cell types, are involved in transporting cholesterol from the outer to the inner mitochondrial membrane in the early stages of steroidogenesis (Sierra, 2004; Garcia-Segura and Melcangi, 2006; Lavaque et al., 2006; Rone et al., 2009). Aromatase is the enzyme involved in the conversion of testosterone to E2, and is expressed in cultured astrocytes and in astrocytes *in vivo* after brain injury (Garcia-Segura et al., 1999; Azcoitia et al., 2003). The expression of TSPO and StAR is also increased after neural injury (Gehlert et al., 1997; Chen and Guilarte, 2008). This increase has been interpreted as an endogenous neuroprotective mechanism, by increasing the local synthesis of neuroprotective steroids, including E2. Our findings, showing that PA reduces the expression of TSPO and aromatase in male astrocytes and increases the expression of StAR in male and female astrocytes, suggest that this FFA may affect steroidogenesis in astrocytes. In addition, TSPO is associated with the control of reactive gliosis (Veiga et al., 2007) and with mitochondrial functions, including the control of apoptosis affecting the inflammatory response (Veenman et al., 2008). The downregulation of TSPO expression by PA may thus contribute to its cytotoxic actions in astrocytes.

Although many studies have suggested that E2 is anti-inflammatory and reduces astrogliosis (Garcia-Estrada et al., 1993) and could prevent endoplasmic reticulum stress in a variety of cells through the inhibition of JNK (Haas et al., 2012), some studies have reported that E2 does not affect CHOP levels (Guo et al., 2014). In the experimental paradigm used here E2 did not elicit an overall protective effect on endoplasmic reticulum stress activated by PA and did not ameliorate the lipoapoptosis. The reported action of estrogens on endoplasmic reticulum stress are mediated by an interaction between E2 and the transcription factor nuclear factor-kappaB (Stice and Knowlton, 2008). However, in our study, the action of PA did not involve this transcription factor.

In attempt to know the fate of the PA, we assessed different enzymes involved in lipid metabolism, observing that PA modifies fatty acid metabolism in cultured astrocytes. Fatty acid levels are determined by the balance between *de novo* synthesis and fatty acid oxidation. The *de novo* synthesis is controlled by ACAC, which leads to the production of malonyl-CoA and FAS, which results in long chain fatty acids that are stored as triglycerides (TG) during energy surplus. Fatty acid oxidation is controlled by CPT1, the rate-limiting enzyme for entry of long-chain acyl-CoAs into the mitochondria during energy deficit (Natali et al., 2007). We found no differences in the mRNA levels of ACAC or CPT1. Interestingly, PA increased the mRNA levels of FAS in astrocytes from males, suggesting that an excess of PA induces lipogenesis only in astrocytes from males. This stimulation of FAS, without an effect on ACAC or CPT1, could be a mechanism to reduce PA-induced lipotoxicity. Studies in β-cells shows that the stimulation of lipid metabolism, including lipogenesis and fatty acid oxidation, protected β-cells from PA-induced lipotoxicity and that protection through enhanced lipogenesis was likely due to reduced ER stress (Choi et al., 2011). A more exhaustive study, including the study of enzymatic activity, is needed to provide a better understanding of the role of PA in lipogenesis in astrocytes. The role of LPL in the brain is to convert TG-rich lipoproteins into FA locally (Wang and Eckel, 2012). PA did not affect levels of LPL mRNA, but normalized the levels of LPL that tended to increase, although not statistically significant, in response to E2 in males.

One caveat that should be taken into consideration when interpreting these studies is the use of ethanol as a solvent for PA and E2. Although this method of treatment preparation has been widely used in the literature, and the same concentration of the solvent was added to the control cultures, we cannot rule out the possibility that the ethanol employed modified the basal state of the astrocytes.

## Conclusion

Our study shows that an increase in saturated fatty acids is a lipotoxic stimulus for glial cells and this could be involved in brain inflammation and endoplasmic reticulum stress in the hippocampus in situations of increased lipids, such as in obesity. Moreover, although E2 induced some protective pathways, it was not able to reverse the lipotoxic effect of PA. The sexually dimorphic differences observed in astrocytes from males and females could be involved in the sex differences in the propensity to develop cognitive dysfunction.

## Author Contributions

LF, LG-S, and JC conceived and designed the experiments. LF and SC performed the experiments. LF, JC, VB, AF-R, PA-A, LG-S, and JA analyzed the data. LF and JC wrote the paper.

## Conflict of Interest Statement

The authors declare that the research was conducted in the absence of any commercial or financial relationships that could be construed as a potential conflict of interest.
