# Supplementary material for: Estradiol Uses Different Mechanisms in Astrocytes from the Hippocampus of Male and Female Rats to Protect against Damage Induced by Palmitic Acid
Source: Front Mol Neurosci. 2017 Oct 24;10:330. doi: 10.3389/fnmol.2017.00330 (PMC5660686; doi:10.3389/fnmol.2017.00330)
Supplement: Supplementary file 1 [file Table_1.DOCX]

**Table 1. A**ssay-on-demand kits (ThermoFisher) and qPCR Primer assays (Qiagen) used

| **Gen** | **Name of Gen** | **Manufacturer, catalog number** | **Species** |
| --- | --- | --- | --- |
| ER1 | Estrogen receptor alpha | ThermoFisher Scientific; | Rat |
|  |  | Rn 01640372_m1 |  |
| ER2 | Estrogen receptor Beta | ThermoFisher Scientific; | Rat |
|  |  | Rn 00562610_m1 |  |
| cyp19a1 | Aromatase | ThermoFisher Scientific; | Rat |
|  |  | Rn 00567222_m1 |  |
| TSPO | Translocator protein | ThermoFisher Scientific; | Rat |
|  |  | Rn 00560892_:m1 |  |
| STAR | Steroidogenic acute regulatory protein | ThermoFisher Scientific; | Rat |
|  |  | Rn 00580695_m1 |  |
| LPL | Lipoprotein lipase | ThermoFisher Scientific; |  |
|  |  | Rn 00561482_m1 | Rat |
| FAS | Fatty acid synthase | ThermoFisher Scientific; |  |
|  |  | Rn 00569117_m1 | Rat |
| ACAC | Acetyl-coA carboxylase | ThermoFisher Scientific; |  |
|  |  | Rn 00573474_m1 | Rat |
| CPT1a | Carnitine palmitoyltransferase | ThermoFisher Scientific; |  |
|  |  | Rn 00580702_m1 | Rat |
| GAPDH | Glyceraldehyde 3-phosphate dehydrogenase | ThermoFisher Scientific; | Rat |
|  |  | Rn 99999916-s1 |  |
| IL6 | Interleukin 6 | QIAGEN PPR06483B | Rat |
| TNFa | Tumor necrosis factor alpha | QIAGEN PPR06411F | Rat |
| IL10 | Interleukin 10 | QIAGEN PPR06479A | Rat |
| GAPDH | Glyceraldehyde 3-phosphate dehydrogenase | QIAGEN PPR06557B | Rat |
