# Supplementary material for: Estradiol Uses Different Mechanisms in Astrocytes from the Hippocampus of Male and Female Rats to Protect against Damage Induced by Palmitic Acid
Source: Front Mol Neurosci. 2017 Oct 24;10:330. doi: 10.3389/fnmol.2017.00330 (PMC5660686; doi:10.3389/fnmol.2017.00330)
Supplement: Supplementary file 2 [file Table_2.DOCX]

**Table 2.** Antibodies used

| **Peptide/protein target** | **Name of Antibody** | **Manufacturer, catalog #, and/or name of individual providing the antibody** | **Species raised in; monoclonal or polyclonal** | **Dilution used** |
| --- | --- | --- | --- | --- |
| Akt | Anti-Akt1/2 | Santa Cruz Biotechnology Cat N sc-1619 | Goat, polyclonal | 1:1000 |
| caspase-3 | Anti-Cleaved Caspase-3 (Asp175) | Cell Signaling Cat N 9661 | Rabbit; polyclonal | 1:500 |
| CHOP | Anti-GADD | Santa Cruz Biotechnology Cat N sc-7351 | Mouse; monoclonal | 1:1000 |
| eIF2α | Anti-eIF2α | Santa Cruz Biotechnology Cat N sc-11386 | Rabbit; polyclonal | 1:1000 |
| ERK1/2 | Anti-ERK1/2 | Santa Cruz Biotechnology Cat N sc-135900 | Mouse; monoclonal | 1:1000 |
| GAPDH | Anti-GAPDH | Anaspec cat N 54593 | Rabbit; polyclonal | 1:1000 |
| GFAP | Anti-glial fibrillary acidic protein (GFAP) | Sigma CatN G3893 | Mouse; polyclonal | 1:5000 |
| HSP70 | HSP70/HSP72 | ENZO Life Sciences ADI-SPA-812-F | Rabbit; polyclonal | 1:1000 |
| IL-6 | Rat IL-6 | R&D Systems Cat N AF506 | Goat; polyclonal | 1:1000 |
| JNK | JNK1(F-3) | Santa Cruz Biotechnology Cat N sc-1648 | Mouse; monoclonal | 1:1000 |
| p38 | p38 α/β (A−12) | Santa Cruz Biotechnology Cat N sc-7972 | Mouse; monoclonal | 1:1000 |
| p-p38 | Anti-Active p38 pAb (pTGpY) | Promega; V121A | Rabbit; polyclonal | 1:1000 |
| p-Akt (ser473) | Anti-Phospho-Akt (Ser473) 587F11 | Cell Signaling Cat N 4051 | Mouse; monoclonal | 1:1000 |
| p-eIF2α | Anti-p-eIF2α (Ser51) | Cell Signaling Cat N 9721 | Rabbit; polyclonal | 1:1000 |
| p-ERK (Thr202/Tyr204) | Anti-Phospho-p44/42 MAPK (ERK1/2) (Thr202/Tyr204) | Cell Signaling Cat N 9101 | Rabbit; polyclonal | 1:1000 |
| p-IκB (Ser 32) | Anti-phospho-IκB-α (Ser32) | Cell Signaling Cat N 9241 | Rabbit; polyclonal | 1:1000 |
| p-JNK | Anti Active JNK pAB (pTPpY) | Promega; V7932 | Rabbit; polyclonal | 1:1000 |
| Vimentin | Anti- vimentin C-terminal antibody | Sigma CatN SAB4503083 | Rabbit; polyclonal | 1:1000 |
